# MOF Derivatives Confined Within Self-Supporting Bamboo Substrates with Hierarchical Porous Architectures for Long-Term Cycling Stability in Zinc–Air Batteries

**DOI:** 10.3390/ma19030598

**Published:** 2026-02-04

**Authors:** Yating Guo, Ailing Feng, Yue Peng, Xing Liu, Shebao Lin, Peitao Liu, Yanqing Zu, Xiaodong Li

**Affiliations:** Key Laboratory of Materials Physics and Functional Devices of Baoji, Institute of Physics and Optoelectronics Technology, Baoji University of Arts and Sciences, Baoji 721016, China; guoyating@stu.bjwlxy.edu.cn (Y.G.); pengyue@stu.bjwlxy.edu.cn (Y.P.); liuxing@stu.bjwlxy.edu.cn (X.L.); zuyanqing74@bjwlxy.edu.cn (Y.Z.); lixxxd@163.com (X.L.)

**Keywords:** MOF derivatives, bifunctional oxygen electrocatalysis, bamboo substrates, self-supporting, zinc–air batteries

## Abstract

**Highlights:**

**What are the main findings?**

- A self-supported Co-N@CB electrocatalyst was prepared using bamboo-derived porous carbon and ZIF-67, integrating biomass sustainability with MOF-derived Co-N sites.
- The catalyst retains a hierarchical porous structure, providing abundant accessible Co-N active sites for ORRs and OERs.
- Co-N@CB showed a low OER overpotential (1.5 V) and excellent zinc–air battery performance (Δ*E* = 0.69 V, >10,000 cycles).

**What are the implications of the main findings?**

- Bamboo-derived carbon offers a binder-free, self-supported platform for high-performance electrocatalysts.
- The hierarchical porous network facilitates mass transport and active site accessibility.
- The long-term stability and high efficiency of Co-N@CB highlight its promise as a biomass-derived cathode for zinc–air batteries.

**Abstract:**

The relatively poor cycle stability of zinc–air batteries (ZABs) hinders their widespread application, while self-supporting electrode materials have shown great potential in enhancing the cycling stability of ZABs. To construct a self-supporting electrode, bamboo was employed as a sustainable precursor, and a two-step pyrolysis strategy was implemented to integrate ZIF-67-derived catalysts onto a hierarchically porous carbon framework, yielding the composite material Co-N@CB. Benefiting from its structural and electronic advantages, Co-N@CB exhibits outstanding electrocatalytic performance. The overpotential for the oxygen evolution reaction (OER) in alkaline electrolyte is 1.5 V at 10 mA cm^−2^, with a potential gap (Δ*E*) of 0.69 V. This material is directly used as the air cathode in ZABs, delivering over 10,000 stable cycles. This excellent cycling stability arises from the strong carbon framework provided by bamboo and the enhanced electrical conductivity achieved through the pyrolytic graphitization of ZIF-67. This study paves the way for further exploration of biomass-based self-supporting electrodes toward high-performance ZABs and emerging micro/nanoscale sensing technologies.

## 1. Introduction

As the global economy continues to advance, the energy crisis and environmental pollution have emerged as pressing challenges facing humanity. To ensure sustainable energy development in the future, it is essential to develop novel, green, and highly efficient energy storage devices. Among various energy storage systems, zinc–air batteries (ZABs) are considered highly promising due to their high theoretical energy density, environmental friendliness, safety, and low cost [1]. However, the sluggish kinetics of both oxygen reduction reactions (ORRs) and oxygen evolution reactions (OERs) significantly limit the power density, efficiency, and cycle stability of ZABs [2,3,4].

In contemporary research, Pt-based catalysts are widely regarded as the benchmark for ORRs, while Ir-based materials (including IrO_2_) and RuO_2_ are commonly employed as reference OER catalysts owing to their outstanding catalytic performance [5,6,7,8]. However, owing to factors such as limited resource reserves, high costs, and insufficient stability, precious metal catalysts are difficult to achieve large-scale application in the field of electrochemical energy conversion [9]. Composite electrocatalysts of transition metals and carbon materials have attracted significant attention due to their abundance, low cost, and tunable structures, finding wide application in various electrocatalytic processes [10,11,12,13,14]. Nevertheless, their electrocatalytic activity and operational stability still require further enhancement owing to insufficient active sites and slow mass transport kinetics. The development of metal-loaded carbon with porous and hierarchical structures can significantly enhance catalytic activity and stability by improving the utilization of catalytic centers and optimizing mass transfer [15]. Moreover, the self-supporting architecture eliminates the need for binders and current collectors, simplifying electrode fabrication. It also reduces interfacial resistance, which enhances electron and ion transport and contributes to improved long-term cycling stability [16]. By combining this structural advantage with the intrinsic porosity and sustainability of biomass materials, the resulting catalysts achieve excellent structural tunability and functional versatility [17]. Such integration provides a novel pathway for the development of efficient and environmentally friendly electrocatalytic systems.

The natural biomass material bamboo has attracted considerable research interest owing to its abundant cellulose, hemicellulose, and lignin content and its well-organized porous structure. The hollow tubular cavities promote efficient fluid transport, while the micrometer-scale vascular bundle pores significantly increase the specific surface area [18]. Upon carbonization, these structural features evolve into a multi-level pore network that synergistically optimizes oxygen diffusion channels and electrolyte penetration. The high aspect ratio fiber network also provides a stable base for catalyst loading, and its natural conductivity, after modification, may enhance the charge transfer efficiency. Cui et al. [19] developed N-doped biomass-derived porous carbon air cathodes for ZABs, achieving a peak power density of 249 mW cm^−2^ and stable operation for 300 h. However, the study did not demonstrate OER performance and evaluated charge–discharge cycling only at low current densities, indicating that further improvements are needed.

Metal–Organic Frameworks (MOFs) are porous crystals assembled via coordination bonds bridging metal-based nodes and organic linkers [20]. Its highly ordered periodic structure enables uniform distribution of active sites throughout the entire framework, which facilitates efficient catalytic reactions. Benefiting from their well-defined structural order, abundant active sites, rapid mass transfer, and tunable porosity, MOFs have shown great potential as non-noble metal electrocatalysts [21]. In particular, ZIF-67 possesses a large surface area and uniform micropores, promoting substance diffusion, increasing active sites, and improving catalytic and energy storage performance, thereby gaining significant interest. He et al. [22] transformed ZIF-67 into CoNi-decorated hollow carbon frameworks via templating, achieving remarkable electrocatalytic performance in both ORRs and OERs. However, most of the electrocatalysts based on ZIF-67 exist in powder form. In practical energy devices, polymer binders and conductive additives inevitably induce catalyst heterogeneity and hinder charge and mass transport across the electrode [23,24,25]. To overcome these limitations, self-supporting electrodes that eliminate the need for polymer binders and conductive additives have attracted considerable attention. In alignment with this strategy, embedding ZIF-67 into the natural pores of bamboo is expected to yield high-performance catalysts suitable for the development of zinc–air batteries.

In this work, a bifunctional oxygen electrocatalyst (Co-N@CB) with highly dispersed Co nanoparticles was successfully synthesized by further carbonizing the ZIF-67-loaded carbonized bamboo (CB) composite. An alkaline treatment with KOH solution was applied to bamboo to remove impurities. This process also increased the pore volume and porosity, facilitating electrolyte penetration and mass transfer. The incorporation of ZIF-67 introduced more active sites, significantly improving the catalytic performance for both ORRs and OERs, with an *E*_1/2_ of 0.81 V and an OER overpotential of 1.5 V at 10 mA cm^−2^. The as-prepared bamboo-derived self-supporting electrode composite was directly deployed as the air cathode in ZABs, demonstrating 785 mAh g^−1^ specific capacity over 10,000 stable cycles for practical energy storage. This study provides novel insights into the utilization of bamboo-derived biomass for advanced energy storage and conversion systems.

## 2. Materials and Methods

### 2.1. Preparation of CB

Cut the bamboo (Baoji University of Arts and Sciences, Baoji, China) raw materials into bamboo slices with dimensions of 1 cm (transverse direction) × 4 cm (longitudinal direction) × 0.25 cm (vertical direction). After soaking in 10% KOH (Aladdin, Shanghai, China) for 12 h, the bamboo slices underwent sequential washing with deionized water and anhydrous ethanol, then were oven-dried at 80 °C for 12 h. Subsequently, the dried samples were heated to 800 °C at a ramping rate of 5 °C min^−1^ under an N_2_ atmosphere for 2 h to achieve pyrolysis, yielding carbonized bamboo (designated as CB).

### 2.2. Preparation of Co-N@CB

6.552 g of Co(NO_3_)_2_·6H_2_O (Aladdin, Shanghai, China) was mixed with 50 mL methanol (Macklin, Shanghai, China) under magnetic agitation for 20 min, ultimately producing a homogeneous cobalt-containing liquid phase. Meanwhile, 2-Methylimidazole (7.938 g. Aladdin, Shanghai, China) was dispersed in 100 mL methanol and continuously stirred for 20 min to prepare the ligand solution. The cobalt salt solution was added dropwise to the obtained ligand solution and allowed to react for 4 h. After solid–liquid separation, ZIF-67 was obtained. Subsequently, add the obtained ZIF-67 to 30 mL of methanol to form an impregnation solution. CB was immersed in the aforementioned solution with 24 h of soaking. The sample was placed in a drying oven at 60 °C for 12 h. Subsequently, it was pyrolyzed at 900 °C under a nitrogen atmosphere with a heating rate of 5 °C min^−1^. After maintaining this temperature for 2 h, the CB composite material (denoted as Co-N@CB) was obtained.

## 3. Results

### 3.1. Composition and Structure Characterizations

**Figure 1:**
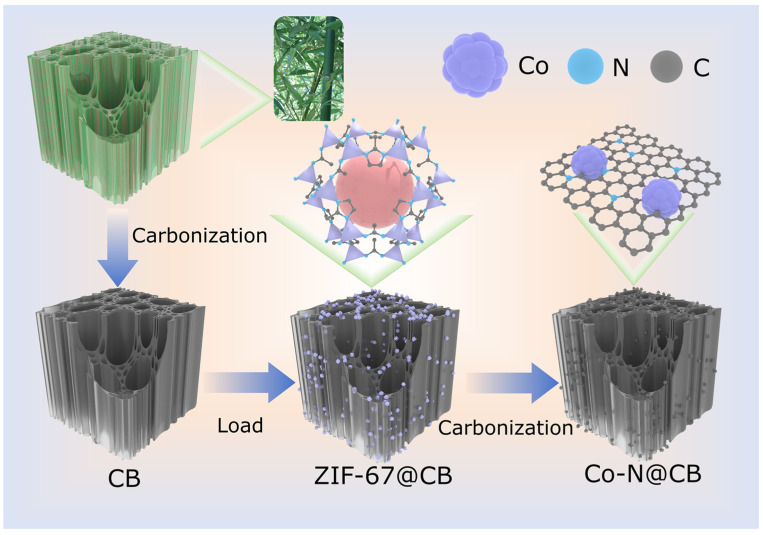
Schematic illustration for the synthetic process of Co-N@CB.

As illustrated in Figure 1, natural bamboo was immersed in a 10% KOH solution for partial lignin removal and then pyrolyzed at 800 °C to obtain carbonized bamboo (CB). Subsequently, the obtained CB was immersed in a methanol solution containing cobalt nitrate and 2-methylimidazole. After ultrasonic immersion, the Co-ZIF particles were uniformly loaded onto the 3D porous network of CB (ZIF-67@CB). ZIF-67@CB was directly carbonized at 900 °C in N_2_ atmosphere to obtain Co-N@CB.

**Figure 2:**
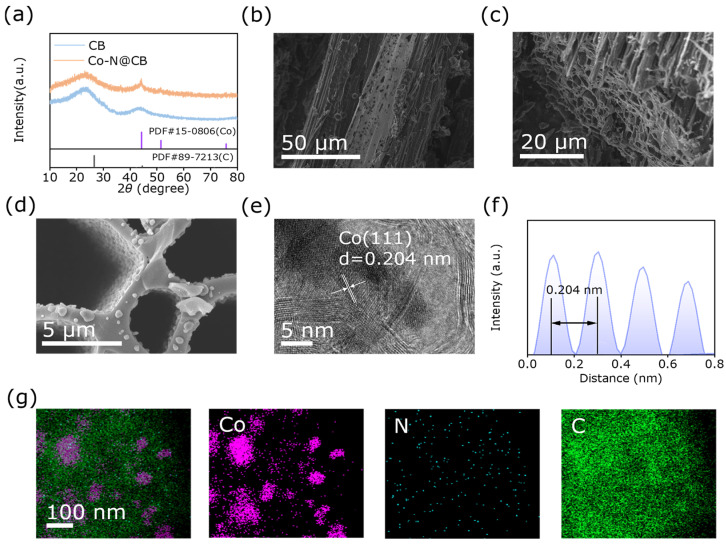
(**a**) XRD patterns of CB and Co-N@CB. SEM images of (**b**) vertical-type CB, (**c**) parallel-type CB, and (**d**) Co-N@CB. (**e**) HRTEM images of Co-N@CB (The arrow in the figure indicates the lattice spacing). (**f**) The intensity distribution map of Co. (**g**) EDS-STEM elemental mapping images of Co-N@CB.

The phase composition and crystalline structure characteristics of Co-N@CB and CB were analyzed by X-ray diffraction (XRD. Bruker D8, Karlsruhe, Germany). As depicted in Figure 2a, the CB sample exhibits characteristic amorphous carbon peaks at 26° and 45°. After loading ZIF-67 onto the sample, a new diffraction peak emerges at 44.2°, which can be indexed to the (111) plane of metallic Co (PDF #15-0806) [26]. This indicates the formation of elemental Co during pyrolysis. Scanning electron microscopy (SEM, Hitachi Flex-2000, Tokyo, Japan) and transmission electron microscopy (TEM, JEOL JEM 2100F, Tokyo, Japan) were employed to characterize the microstructure and morphology of the sample. As illustrated in Figure 2b,c, the longitudinal and transverse cross-sections of the CB reveal a graded porous structure, which enhances its specific surface area and mass transfer efficiency [27]. In contrast, Figure S1 shows the pristine CB derived from raw bamboo without KOH treatment, exhibiting a relatively smooth surface with no obvious pores. Figure S2 indicates the successful and uniform loading of ZIF-67 within the channels of the CB. The cross-sectional view in Figure S3 further illustrates its well-defined embedding, highlighting the uniform dispersion of ZIF-67 throughout the internal structure. After pyrolysis, as shown in Figure 2d, CB still maintained its original porous structure with a rough surface and a 3D network. In contrast, the ZIF-67 loaded within the bamboo pores exhibited a slight morphological collapse. Subsequently, SEM characterization was performed on the sample derived from raw bamboo without KOH treatment and subsequently loaded with ZIF-67 followed by carbonization (Co–N@CB (pristine)), as shown in Figure S4. Serving as a precursor, ZIF-67 decomposed during pyrolysis to form Co-containing active species. These species are confined within the carbon pores, effectively preventing their migration and aggregation, thereby enhancing the structural stability and service life of the catalyst. TEM and high-resolution transmission electron microscopy (HRTEM) characterization verified the successful synthesis regarding Co-N@CB. The interplanar spacing of Co-N@CB was 0.204 nm, matching the crystal plane of Co (111) (Figure 2e,f and Figure S5). Energy-dispersive X-ray spectroscopy (EDS, JEOL JEM 2100F, Japan) mapping confirmed the uniform distribution of C and N, as well as Co nanoparticles, throughout the Co-N@CB matrix (Figure 2g).

**Figure 3:**
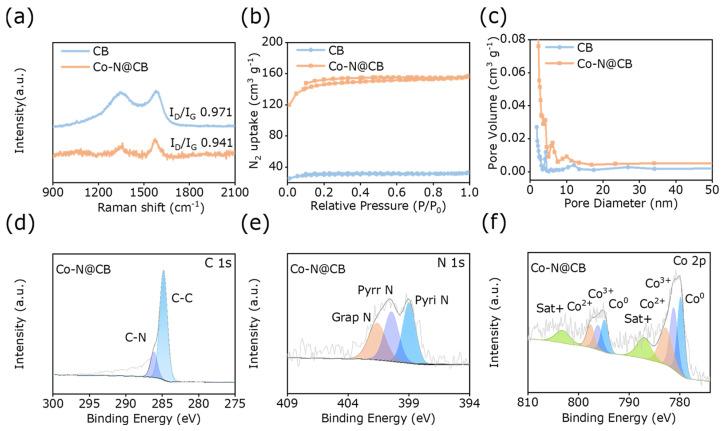
(**a**) Raman spectra, (**b**) N_2_ adsorption–desorption isotherms, and (**c**) pore size distribution curves of CB and Co-N@CB. High-resolution (**d**) C 1s, (**e**) N 1s, (**f**) Co 2p XPS spectra for Co-N@CB.

CB and Co-N@CB exhibit D bands (1355 cm^−1^) and G bands (1595 cm^−1^) in their Raman spectra (Figure 3a). Typically, the D band is associated with structural defects in the carbon lattice, while the G band corresponds to the planar vibrational characteristics of sp^2^-bonded carbon [28]. The *I_D_*/*I_G_* ratio serves as a quantitative indicator for defect density characterization in materials [29]. The strength ratios of CB and Co-N@CB are 0.971 and 0.941, demonstrating that Co incorporation enhances carbon crystallinity while decreasing lattice defects. The high degree of graphitization is the key to conductivity [30]. Raman spectroscopy after electrochemical testing exhibited an *I_D_*/*I_G_* ratio of 0.948, indicating relatively small structural changes, as shown in Figure S6. N_2_ physisorption measurements were employed to characterize the porous characteristics and Brunauer–Emmett–Teller (BET) surface area of the samples. The nitrogen sorption isotherms of CB and Co-N@CB both display typical Type IV behavior, indicating the presence of micropores and mesopores [31]. The specific surface area of CB is 99.25 m^2^ g^−1^, lower than that of Co-N@CB (469.24 m^2^ g^−1^), demonstrating a substantial increase through cobalt modification (Figure 3b). By comparison, the carbon derived from raw bamboo without KOH treatment and subsequently loaded with ZIF-67 and carbonized (Co-N@CB (pristine)) exhibits a much lower surface area of 60.11 m^2^ g^−1^ (Figure S7). The decrease in specific surface area is likely due to the partial blockage of micropores by Co nanoparticles [32]. Figure 3c shows the pore size distribution of the samples, where Co-N@CB (0.24 cm^3^ g^−1^) has a higher pore volume than CB (0.05 cm^3^ g^−1^). High specific surface area exposes abundant active sites, while high pore volume facilitates reactant diffusion, thereby accelerating redox kinetics. X-ray photoelectron spectroscopy (XPS, Shimadzu Kratos AXIS Supratm, Kyoto, Japan) was conducted to investigate the chemical states, surface composition, doping type, and elemental bonding configuration of Co-N@CB. The results further confirmed that the prepared catalyst samples contained C, N, and Co elements. Figure 3d presents the high-resolution C 1s XPS spectrum of Co-N@CB, which can be deconvoluted into two peaks assigned to C=C (284.8 eV) and C–C/C–N (286.12 eV). Then, an analysis was conducted on species N. Figure 3e shows the deconvoluted N 1s spectrum, which reveals three types of nitrogen configuration including pyridinic (398.97 eV), pyrrolic (400.42 eV) and graphitic nitrogen (401.6 eV) [33]. Pyridine nitrogen and graphite nitrogen are also beneficial for enhancing the activity of ORRs [34]. In the high-resolution Co 2p spectrum, it can be decomposed into Co^0^ (779.79 eV, 794.83 eV), Co^3+^ (781.16 eV, 796.12 eV), Co^2+^ (782.74 eV, 797.59 eV), and satellite (787.04 eV, 803.14 eV) peaks (Figure 3f) [35]. XPS analysis of Co-N@CB after electrochemical testing shows that the chemical states of Co and N remain largely unchanged (Figure S8), indicating that the material maintains its compositional stability.

### 3.2. Electrocatalytic Performances

**Figure 4:**
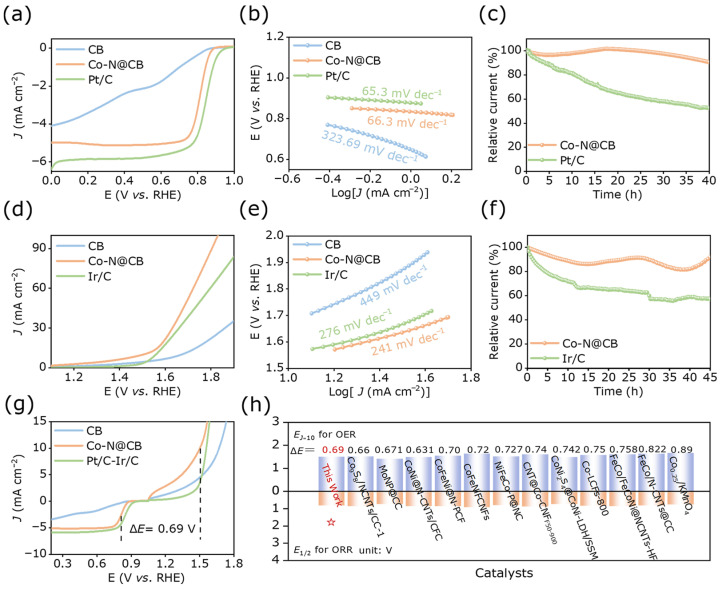
(**a**) The ORR LSV curves of CB, Co-N@CB, and Pt/C in an oxygen-saturated 0.1 M KOH electrolyte at a scanning rate of 2 mV s^−1^. (**b**) The associated Tafel slope. (**c**) The ORR stability of Co-N@CB and Pt/C. (**d**) The OER LSV curves of CB, Co-N@CB, and Ir/C. (**e**) The corresponding Tafel slope. (**f**) The OER stability of Co-N@CB and Ir/C. (**g**) The bifunctional ORR/OER curves of CB, Co-N@CB, and Pt/C-Ir/C. (**h**) The Δ*E* comparison between Co-N@CB and reported catalysts, and the red star indicates the results of this work.

The ORR performance is related to the discharge process of ZABs. We evaluated the electrocatalytic ORR activity of CB, Co-N@CB, and Pt/C in 0.1 M KOH with O_2_ saturation at a rotation speed of 1600 rpm. The LSV curve in Figure 4a and Figure S9 shows that the *E*_1/2_ of CB is 0.68 V, indicating poor catalytic performance for the ORR. Compared with CB, the Co-N@CB catalyst exhibits improved ORR activity, showing an *E*_1/2_ of 0.81 V, which is marginally below the value observed for Pt/C (0.84 V). The significant oxygen reduction reaction (ORR) activity of Co-N@CB is further evidenced by its smaller Tafel slope. This value (66.3 mV dec^−1^) is lower than that of CB (323.69 mV dec^−1^) and slightly higher than that of Pt/C (65.3 mV dec^−1^), which also evidences enhanced charge transfer dynamics in ORRs (Figure 4b). The electron transfer number was determined from LSV measurements at different scan rates using the Koutecky–Levich equation (Figures S10 and S11). The average electron transfer numbers of CB and Co-N@CB are 3.53 and 3.90, respectively. This suggests that Co-N@CB approaches the ideal 4e^−^ ORR pathway, resulting in enhanced reaction selectivity and catalytic performance [36]. The four-electron selectivity of the ORR was evaluated by measuring H_2_O_2_ production using a rotating ring-disk electrode (RRDE) [37]. Figure S12a,b shows that Co-N@CB exhibits a relatively low H_2_O_2_ yield during the ORR process. Its electron transfer number is approximately 3.9, indicating that most oxygen molecules are reduced to H_2_O. This behavior is beneficial for the performance of ZABs [38,39]. Furthermore, after 40 h of stability testing, Co-N@CB maintained a current retention rate of 91%, significantly outperforming Pt/C (53%), demonstrating excellent catalytic durability (Figure 4c).

OER measurements of the fabricated catalysts were performed in alkaline solution under ambient conditions employing a standard three-electrode system. The prepared CB, Co-N@CB samples, and benchmark Ir/C were evaluated in a 1 M KOH electrolyte. Firstly, the LSV curves were measured to assess the OER activity of the samples. The Co-N@CB catalyst exhibited excellent OER activity and favorable kinetics, requiring only 1.5 V to achieve 10 mA cm^−2^, which is significantly reduced compared to CB (1.67 V) and commercial Ir/C (1.55 V) (Figure 4d and Figure S13). Subsequently, the Tafel slopes for the samples were derived through linear fitting. The lower Tafel slope of Co-N@CB (241 mV dec^−1^) indicates superior reaction kinetics compared to CB (449 mV dec^−1^) and commercial Ir/C (276 mV dec^−1^) (Figure 4e). Electrochemical impedance spectroscopy (EIS) reveals that Co-N@CB exhibits a lower charge transfer resistance than CB and commercial Ir/C, indicating enhanced OER kinetics and a more efficient electron transfer process (Figure S14) [40]. The inclined line at low frequencies is attributed to Warburg-type diffusion impedance, reflecting mass transport of reactants and products within the porous catalyst layer and the electrolyte [41]. CB exhibits a pronounced diffusion feature, indicating relatively hindered mass transport, whereas Co-N@CB shows no distinct diffusion tail, suggesting improved surface accessibility and interfacial transport favorable for the electrochemical reactions. The Rct values and corresponding literature data are summarized in Table 1 for comparison. Next, the CV curves obtained under different sweep rates were used to obtain the double-layer capacitance (*C_dl_*) of different samples (Figures S15 and S16). The electrochemical active surface area (ECSA) exhibits a linear dependence on the *C_dl_* and serves as a key metric for assessing catalytic activity [42]. The double-layer capacitance, *C_dl_*, was calculated from Δi vs. ν plots constructed from CV curves, following previously reported methods [43]. The *C_dl_* value was determined to be 28.14 mF for Co-N@CB, slightly higher than the corresponding values of Ir/C (26.18 mF) andCB (5.52 mF). This indicates that Co-N@CB has a larger ECSA, effectively exposing more active sites and thus exhibiting higher catalytic activity. For reference, the *C_dl_* values are compared with representative literature data and summarized in Table S1. This further indicates that Co-N@CB has faster kinetics and has more active sites in its porous carbon structure.

In addition to the aforementioned high catalytic activity, chronoamperometric experiments demonstrated that after a 45-h stability test, the current retention rate of Co-N@CB was 92%, whereas that of Ir/C was only 57% (Figure 4f). This result indicates that the Co-N@CB catalyst has excellent OER stability. Then calculate the potential difference (Δ*E*) between *E_j_*_= 10_ for the OER and *E*_1/2_ for the ORR from the LSV curves to evaluate the bifunctionality of the catalyst. Co-N@CB had the smallest potential difference Δ*E* of only 0.69 V (Figure 4g). Compared with most recently reported catalysts, the Co-N@CB catalyst exhibits relatively better electrocatalytic activity for ORRs/OERs (Figure 4h, Table S2), demonstrating its potential for application in ZABs [44,45,46,47,48,49,50,51,52,53,54,55].

**Table 1:** Comparison of Charge Transfer Resistance (Rct) for Samples with Literature Data.

Sample Name	Rct (Ω)	Electrolyte	References
CB Co-N@CB	20.38 6.69	1M KOH	This work
DNiFe LDH-2h@CoNi-NCNT	0.22	1M KOH	[56]
Co@TC-0.4	3.23	1M KOH	[57]
CC@FeCoNiMoRu-HEA/C	7.6	1M KOH	[58]
3NiFe-1N-GA-800	10	1M KOH	[59]
N-Fe@6 NPs	15	1M KOH	[60]
4CeO_2_@SrIrO_3_	22.6	0.5M H_2_SO_4_	[61]
P-MnCo_2_O_4_@PWC	26	0.1M KOH	[62]
IrO_2_/GS	47	1M KOH	[63]
CoNiLDH@NPC	52.2	1M KOH	[64]
NiFe/NC-900	123.6	1M KOH	[65]

### 3.3. Application Analysis of ZABs

**Figure 5:**
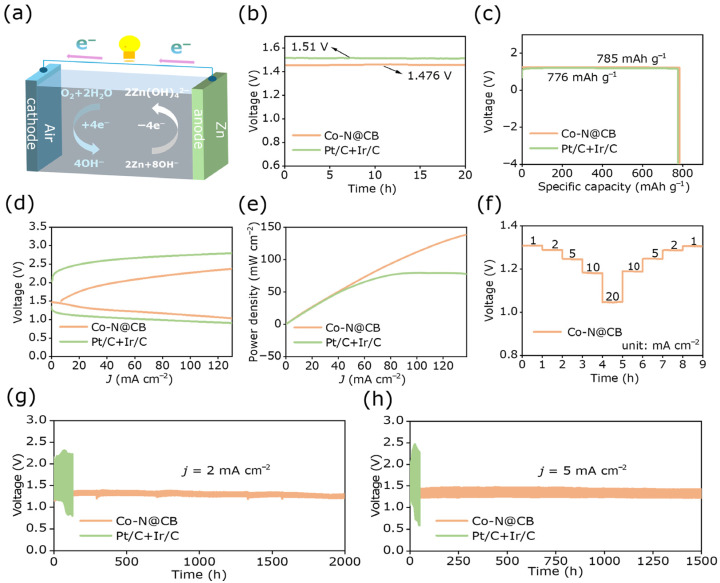
(**a**) Schematic diagram of liquid ZAB. (**b**) Open-circuit voltage curve, (**c**) Specific capacity, (**d**) Discharge polarization curve, and (**e**) Corresponding power density of Co-N@CB and Pt/C + Ir/C. (**f**) Voltage profiles of Co-N@CB at different current densities. (**g**,**h**) long-term cycling stability of ZABs based on Co-N@CB and Pt/C + Ir/C.

ZABs are considered promising alternatives for future energy storage solutions, owing to their superior energy density, eco-friendliness, and cost-effectiveness. The practical viability of Co-N@CB as a bifunctional electrocatalyst in ZABs was systematically evaluated through comprehensive tests. Using zinc sheets as the anode, a 6 M KOH + 0.2 M Zn (CH_3_COO)_2_ solution as the electrolyte, and Co-N@CB as the air electrode, a ZAB was constructed (Figure 5a). A control ZAB was assembled noble metal catalysts Pt/C + Ir/C was used as the control experiment.

Figure 5b shows that the Co-N@CB-based ZABs deliver an open-circuit voltage of 1.476 V, close to that of Pt/C + Ir/C-based ZABs (1.51 V). Under a current density of 10 mA cm^−2^, the specific capacity of the ZABs assembled based on Co-N@CB was 785 mAh g^−1^, outperforming conventional Pt/C + Ir/C (776 mAh g^−1^) (Figure 5c). According to Figure 5d, Co-N@CB displays a narrower charge–discharge voltage gap compared with Pt/C + Ir/C. This indicates superior charge–discharge characteristics, leading to reduced energy loss and improved voltage stability during cycling. The peak power density reached 138.63 mW cm^−2^, significantly exceeding that of Pt/C + Ir/C, which is 77.8 mW cm^−2^ (Figure 5e). The Co-N@CB-based ZABs show stable discharge voltage plateaus from 1 to 20 mA cm^−2^, demonstrating excellent rate performance and electrochemical stability, which supports long-term battery operation (Figure 5f). Finally, the ZABs assembled by Co-N@CB were subjected to a cycling stability test at a current density of 2 mA cm^−2^. Experimental data demonstrated that after 2000 h, the voltage difference between charging and discharging remained essentially stable (Figure 5g and Figure S17a). The ZABs assembled with Pt/C + Ir/C showed significant voltage gap changes after 50 h. Figure 5h and Figure S17b demonstrate that at 5 mA cm^−2^, the Co-N@CB-based ZABs exhibited stable cycling with a relatively small voltage difference over 1500 h. The experiment was stopped at 1500 h due to an unexpected interruption, and the cell performance remained stable until that point. In contrast, the Pt/C + Ir/C-based ZABs exhibited a larger voltage difference after only 20 h. Notably, the voltage difference in the Co-N@CB-based ZABs was much smaller than that of the Pt/C + Ir/C-based ones. Cycling test results verified that Co-N@CB exhibits good stability. As shown in Figure S18, the Zn–air battery cycled at 2 mA cm^−2^ maintains a relatively stable Coulombic efficiency over 2000 h of cycling, reaching approximately 96% at the end of the test. This behavior indicates good electrochemical reversibility. Charge–discharge cycling of the Co-N@CB (pristine) sample at 2 mA cm^−2^ exhibited a larger voltage difference compared to Co-N@CB (Figure S19), reflecting less favorable electrochemical kinetics without KOH activation. Subsequently, during the charge–discharge cycle test, pictures of the sample detachment every 24 h for Co-N@CB and Pt/C + Ir/C were taken (Figure S20). It was observed that Co-N@CB exhibited minimal detachment, whereas Pt/C + Ir/C showed more significant detachment. These results confirm the good stability of Co-N@CB. Figure S21a,b illustrate that the Co-N@CB catalyst can be effectively used in ZABs to power panels and beads, confirming its feasibility. The excellent performance of Co-N@CB in ZABs demonstrated its promising prospects for industrial deployment.

## 4. Conclusions

In summary, a bamboo-derived carbon-based composite (Co-N@CB) was successfully synthesized and evaluated as an efficient air-electrode material for ZABs. The composite material exhibits favorable bifunctional catalytic activity toward ORRs and OERs, achieving an ORR half-wave potential (*E*_1/2_) of 0.81 V, an OER overpotential of 1.5 V at 10 mA cm^−2^, and a Δ*E* value of 0.69 V. Moreover, the robust structure of the bamboo-derived carbon contributes to the synthesized Co-N@CB excellent durability and cycling stability. As a result, the assembled ZABs demonstrate a cycling stability of 2000 h at a current density of 2 mA cm^−2^ and 1500 h at 5 mA cm^−2^, while delivering a peak power density of 138.63 mW cm^−2^ at 10 mA cm^−2^. These results demonstrate that low-cost and fast-growing biomass-derived bamboo can be effectively utilized to construct durable air electrodes for long-life ZABs. This provides a promising strategy for the development of high-stability and efficient energy conversion catalysts.

## Data Availability

The original contributions presented in this study are included in the article/Supplementary Materials. Further inquiries can be directed to the corresponding authors.

## Supplementary Materials

The following supporting information can be downloaded at: https://www.mdpi.com/article/10.3390/ma19030598/s1. Figure S1: SEM image of CB in the longitudinal direction before KOH treatment; Figure S2: SEM image of the ZIF-67@CB; Figure S3: SEM image of the ZIF-67@CB in the longitudinal direction; Figure S4: SEM image of Co-N@CB (pristine); Figure S5: TEM image of the Co-N@CB; Figure S6: Raman spectra of Co-N@CB measured after a 40 h charging/discharging cycling stability test at 5 mA cm^−2^; Figure S7: BET analysis of Co-N@CB (pristine); Figure S8: High-resolution XPS spectra of Co-N@CB after a 40 h charging/discharging cycling stability test at 5 mA cm^−2^: (a) C 1s, (b) N 1s, and (c) Co 2p regions; Figure S9: The ORR half-wave potentials of CB, Co-N@CB, and Pt/C; Figure S10: (a) LSV curves at different rotation rates (400–2400 rpm) of CB; (b) The corresponding K–L equation graph; Figure S11: (a) LSV curves at different rotation rates (400–2400 rpm) of Co-N@CB; (b) The corresponding K–L equation graph; Figure S12: (a) RRDE voltammograms of Co-N@CB in O_2_-saturated; (b) H_2_O_2_ yields and the corresponding electron transfer number (n) of Co-N@CB; Figure S13: The OER overpotentials of CB, Co-N@CB, and Ir/C; Figure S14: Nyquist curves of CB, Co-N@CB, and Ir/C; Figure S15: Cyclic voltammetry (CV) curves of (a) CB, (b) Co-N@CB, and (c) Ir/C recorded at scan rates of 20, 40, 60, 80, and 100 mV s^−1^ in the non-faradaic potential range of 1.02–1.22 V vs. RHE; Figure S16: The summarized *C_dl_* of CB, Co-N@CB, Ir/C; Figure S17: (a,b) long-term cycling stability of ZABs based on Co-N@CB and Pt/C + Ir/C; Figure S18: Coulombic efficiency of Zn–air batteries based on Co-N@CB measured at a current density of 2 mA cm^−2^ during long-term cycling; Figure S19: Long-term cycling stability of ZABs based on Co-N@CB (pristine) measured at 2 mA cm^−2^; Figure S20: The sample shedding comparison diagram of ZABs based on Co-N@CB under a discharge charge current density of 5 mA cm^−2^ during the cycling test; Figure S21. (a) The beads powered by ZABs assembled with Co-N@CB. (b) The light-emitting diode (LED) panel by ZABs assembled with Co-N@CB. Table S1: Comparison of double-layer capacitance (*C_dl_*) values for different electrocatalysts reported in the literature; Table S2: The electrocatalytic activities of recently reported bifunctional electrocatalysts for OER and ORR. The Supporting Information is available free Experimental details, Electrochemical tests, SEM, TEM, half-wave potentials, LSV RRDE, OER overpotentials Nyquist curves, Cyclic voltammetry, summarized *C_dl_*, cycling stability, and tables. References [44,45,46,47,48,49,50,51,52,53,54,55,66,67,68,69,70,71,72,73,74,75] are cited in the Supplementary Materials.
