# Supplementary material for: MOF Derivatives Confined Within Self-Supporting Bamboo Substrates with Hierarchical Porous Architectures for Long-Term Cycling Stability in Zinc–Air Batteries
Source: Materials (Basel). 2026 Feb 4;19(3):598. doi: 10.3390/ma19030598 (PMC12897813; doi:10.3390/ma19030598)
Supplement: Supplementary file 1 [file materials-19-00598-s001.zip › materials-4062996-supplementary.pdf]

Supporting Information

MOF Derivatives Confined within Self-Supporting Bamboo Substrates with Hierarchical Porous Architectures for Long-Term Cycling Stability in Zinc–Air Batteries

Yating Guo, Ailing Feng*, Yue Peng, Xing Liu, Shebao Lin*, Peitao Liu*, Yanqing Zu, Xiaodong Li

Key Laboratory of Materials Physics and Functional Devices of Baoji, Institute of Physics and Optoelectronics Technology, Baoji University of Arts and Sciences, Baoji 721016, China.

* Corresponding author: E-mail address: ailingfeng@bjwlxy.edu.cn (A. F.); linshebao@163.com (S. Lin.); liupt@bjwlxy.edu.cn (P. Liu.)

Section S1. Experimental details.

1 Raw materials and chemical components

Bamboo [Moso bamboo], Cobalt (II) nitrate hexahydrate [$Co(NO_3)_2·6H_2O$, 99 %] (AR, Aladdin Reagent Co., Ltd.), methanol [C_2H_6O, 99 %] (AR, Macklin Reagent Co., Ltd.), ethanol [C_2H_5OH, 99 %] (AR, Macklin Reagent Co., Ltd.), 2 methylimidazole [$C_4H_6N_2$, 99 %] (AR, Aladdin Reagent Co., Ltd.), potassium hydroxide [KOH, 99 %] (AR, Aladdin Reagent Co., Ltd.), zinc acetate [$C_4H_6O_4Zn·2H_2O$, 99 %] (AR, Aladdin Reagent Co., Ltd.), Nafion-117 [$C_7HF_{13}O_5S$, 5 wt.%] (AR, Dupont USA Co.), N,N-Dimethylformamide [C_3H_7NO, 99 %] (AR, Aladdin Reagent Co., Ltd.), petroleum ether[C_3H_7NO, 99 %] (AR, Aladdin Reagent Co., Ltd.), Pt/C [20 %] (AR, Premetek Co.), Ir/C [20 %] (AR, Premetek Co.). All reagents were directly served as obtained without further depuration.

2 Electrochemical tests

Characterizations: The morphology of the samples were assessed using scanning electron microscopy (SEM; Hitachi Flex-2000, Japan). Transmission electron microscope and High-resolution TEM (TEM and HRTEM; JEOL JEM 2100F, Japan) were used to observe the morphology and structure of the products. Furthermore, we investigated the crystallographic and chemical states of the elements in the samples using powder X-ray diffraction measurement (XRD; Bruker D8, Karlsruhe, Germany) and X-ray photoelectron spectroscopy (XPS; Shimadzu Kratos AXIS Supratm, Japan). Raman spectroscopy was conducted with a Renishaw in Via spectrometer at room temperature. The N_2 adsorption/desorption isotherms were collected at 80 °C by using a Micromeritics ASAP 2460.

Electrochemical measurement: The performance of the prepared catalyst in the alkaline solution of CHI 760E (Shanghai Chen hua Instrument Corporation, Shanghai, China) in the three-electrode system. The platinum sheet was used as the counter electrode and Ag/AgCl as the reference electrode. The rotating disk electrode (RDE) or rotating ring-disk electrode (RRDE) are used as the working electrodes. All the measured potentials

(vs Ag/AgCl) in this work were converted into reversible hydrogen electrodes (RHE), and the iR was corrected according to the Nernst equation ($E_{RHE} = E_{Ag/AgCl} + 0.197 + 0.059pH$). To prepare the catalyst slurry, 3 mg of the sample and 3 mg of carbon black were homogenized in 35 mL petroleum ether via sonication for 4 h. The dispersion was centrifuged and thoroughly dried in an oven at 60 °C. The dried mixture (6 mg) was dispersed in a solution of N, N-dimethylformamide (1470 μL) and Nafion-117 (30 μL), followed by 6 h sonication to form a homogeneous catalyst ink.

The number of electrons transferred (n) and H_2O_2 yield were calculated from the following equations:

$$n = \frac{4 \times I_d}{I_d + I_r/N} \quad [1]$$

$$[H_2O_2]\% = \frac{200 \times I_R/N}{I_R/N + I_D} \quad [2]$$

where Id is the disk current and Ir represents the ring current. N stands for the collection efficiency of the ring electrode (0.40).

Based on LSV curves with various rotating speeds, Koutecky–Levich (K–L) equation can be used to calculate the number of electrons transferred and the mass-transfer corrected kinetic current density J_K.

$$\frac{1}{J} = \frac{1}{J_K} + \frac{1}{J_L} = \frac{1}{J_K} + \frac{1}{B\omega^{1/2}}$$

[3]

$$B = 0.2nFC_0D_0^{2/3}\nu^{-1/6}$$

[4]

Where J and J_L represent measured current density and diffusion-limiting current density, respectively. F is Faraday constant (F = 96485 C mo^{-1}). C_0 (1.2×10^{-6} mol cm^{-3}) and D_0 (1.9×10^{-5} cm^2 s^{-1}, 0.1 M KOH) represent the bulk concentration and diffusion coefficient of O_2, respectively. ν (0.01 cm^2 s^{-1}) is kinetic viscosity. 0.2 is constant when the unit of ω is revolutions per minute (r/min = rpm).

Assembly of liquid zinc–air batteries: The self-made zinc–air battery was tested by using the LAND CT3001A multi-channel battery testing system (manufactured by Wuhan Land Instrument Factory). Zinc foil was adopted as the anode. The prepared self-supported catalyst electrode (Co-N@CB) was directly used as the cathode. For powder samples, the catalyst ink made by mixing Pt/C and Ir/C at a mass ratio of 1:1 was drop-coated onto carbon cloth to form an air electrode, which was then dried at room temperature. The prepared self-supported catalyst electrode (Co-N@CB) was directly used as the cathode. For the powder sample, zinc foil was used as the anode. Then, a zinc–air battery was assembled by filling the electrolyte (6 M KOH and 0.2 M Zn $(Ac)_2$) between the anode and the air cathode, and continuously pumped into the battery.

The specific capacity for Zn–air battery was calculated by the equation below:

$$Specific\ capacity\ (mAh\ g^{-1}) = \frac{I_{dis} \times t}{m} \quad [5]$$

The Coulombic efficiency of the Zn–air battery was calculated using the equation below:

$$Coulombic\ Efficiency(CE) = \frac{Q_{discharge}}{Q_{charge}} \times 100\% \quad [6]$$

where Idis represents the discharge current and t represents the service hours. m denotes the weight of Zn consumed.

Section S2. Supporting figures.

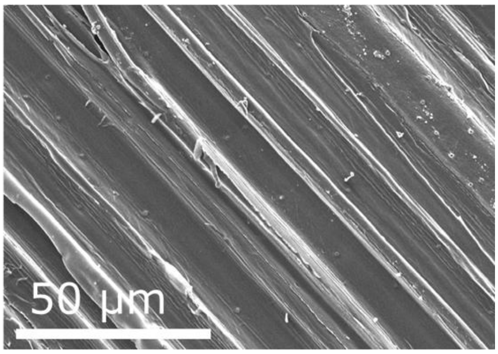

Figure S1. SEM image of CB in the longitudinal direction before KOH treatment.

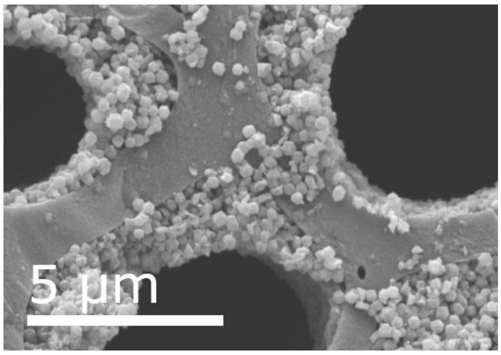

Figure S2. SEM image of the ZIF-67@CB.

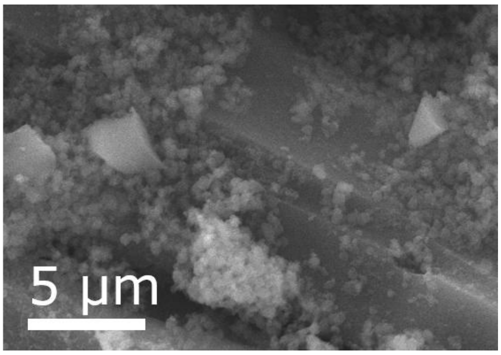

Figure S3. SEM image of the ZIF-67@CB in the longitudinal direction.

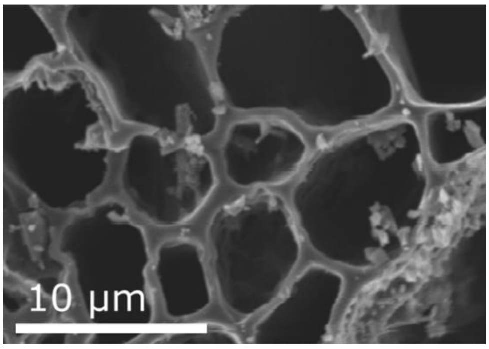

Figure S4. SEM image of Co-N@CB (pristine).

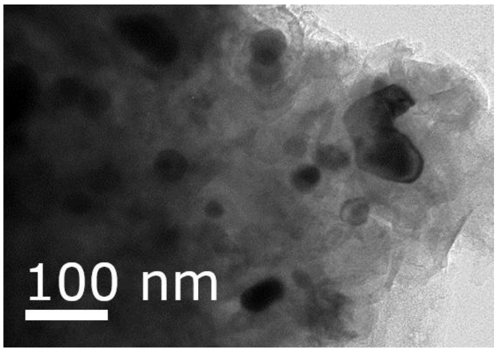

Figure S5. TEM image of the Co-N@CB.

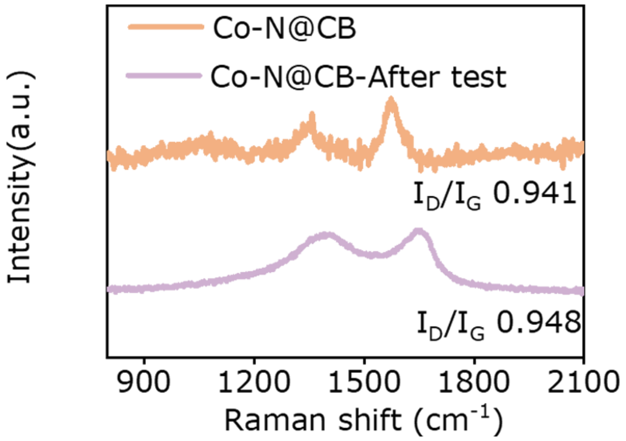

Figure S6. Raman spectra of Co-N@CB measured after a 40 h charging/discharging cycling stability test at 5 mA cm^{-2}.

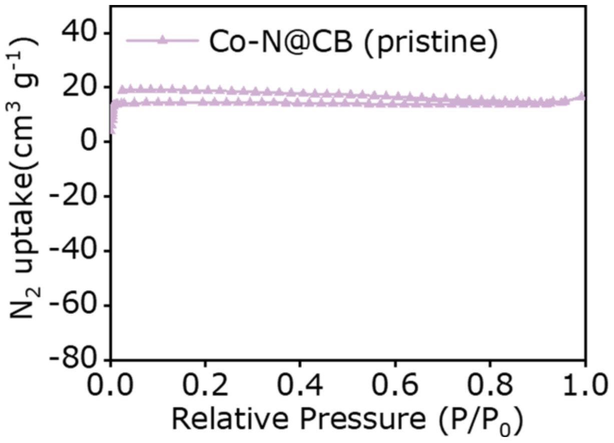

Figure S7. BET analysis of Co-N@CB (pristine).

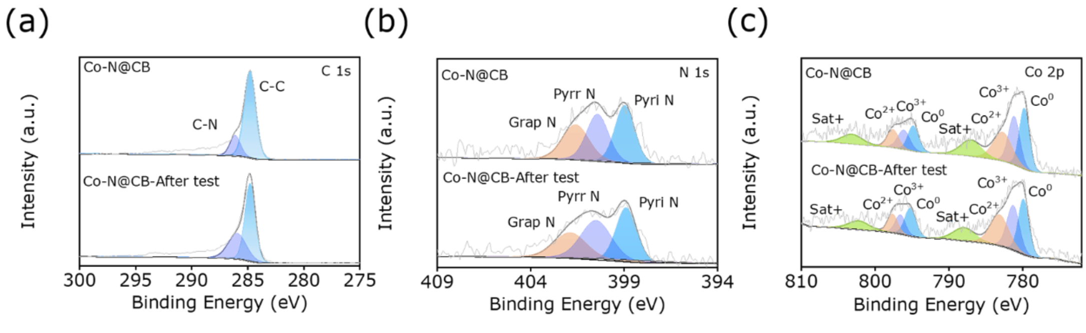

Figure S8. High-resolution XPS spectra of Co-N@CB after a 40 h charging/discharging cycling stability test at 5 mA cm^{-2}: (a) C 1s, (b) N 1s, and (c) Co 2p regions.

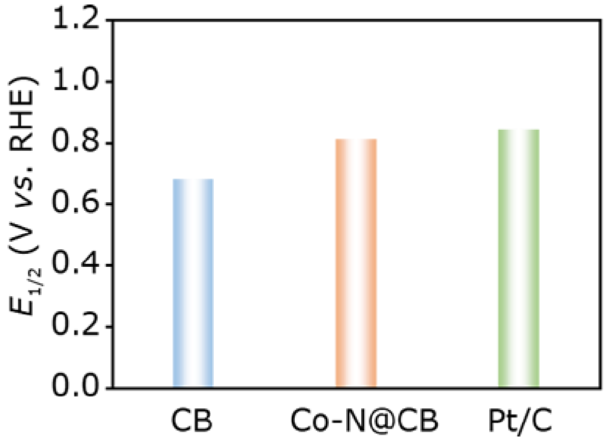

Figure S9. The ORR half-wave potentials of CB, Co-N@CB, and Pt/C.

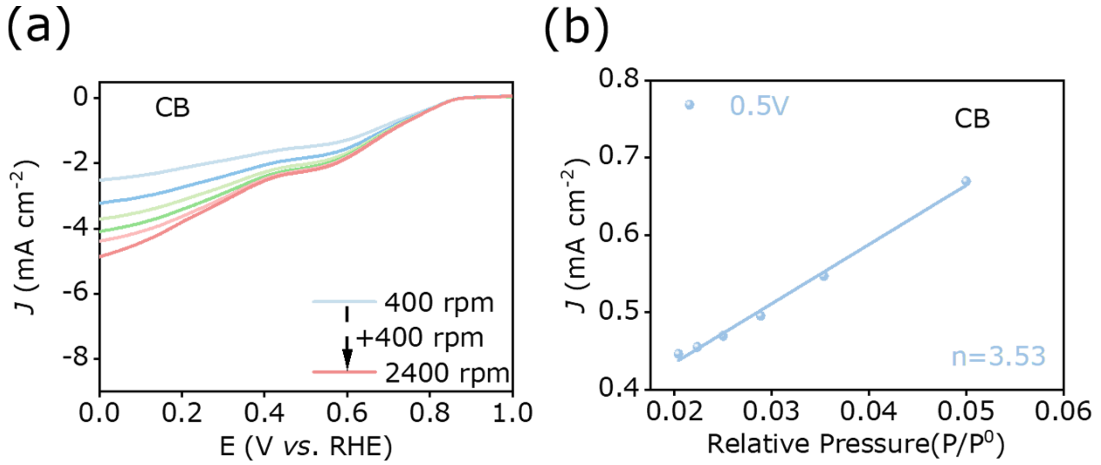

Figure S10. (a) LSV curves at different rotation rates (400–2400 rpm) of CB; (b) The corresponding K–L equation graph.

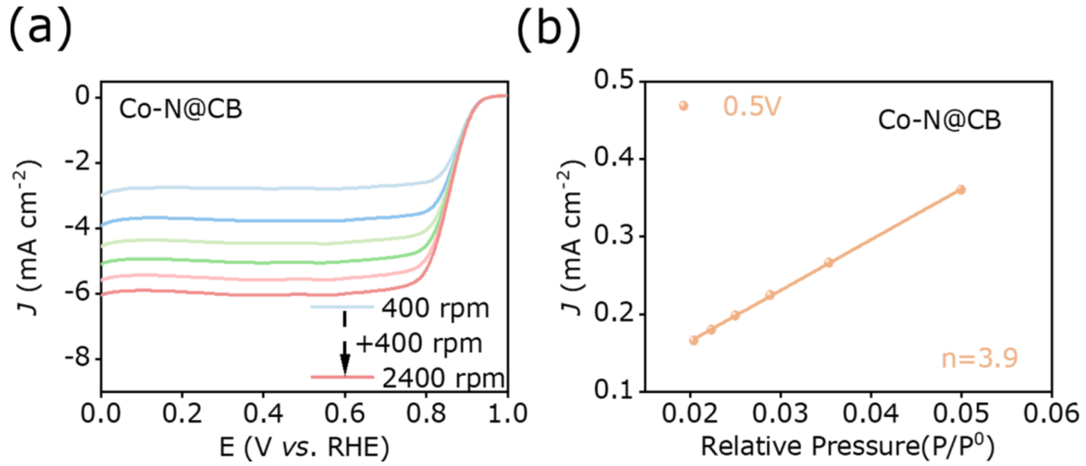

Figure S11. (a) LSV curves at different rotation rates (400–2400 rpm) of Co-N@CB; (b) The corresponding K–L equation graph.

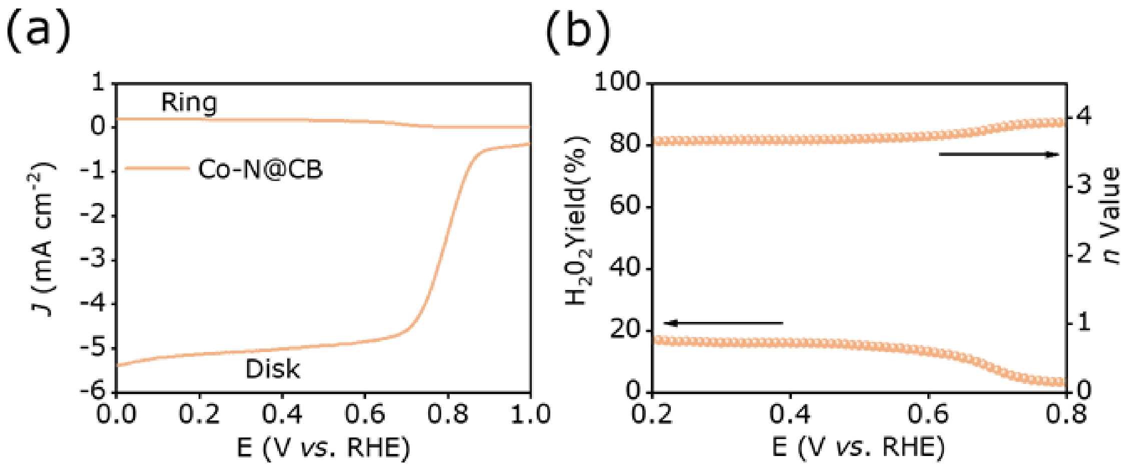

Figure S12. (a) RRDE voltammograms of Co-N@CB in O_2-saturated; (b) H_2O_2 yields and the corresponding electron transfer number (n) of Co-N@CB.

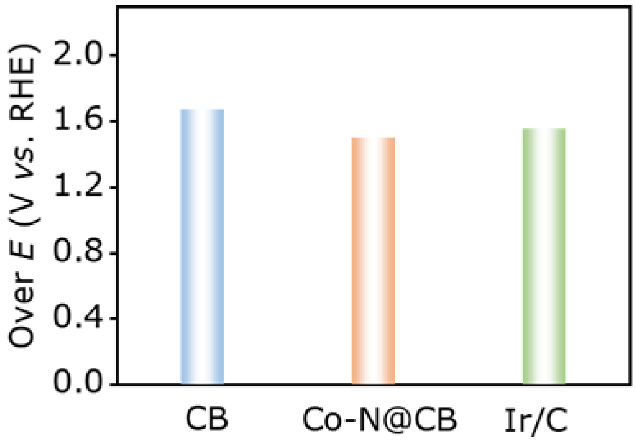

Figure S13. The OER overpotentials of CB, Co-N@CB, and Ir/C.

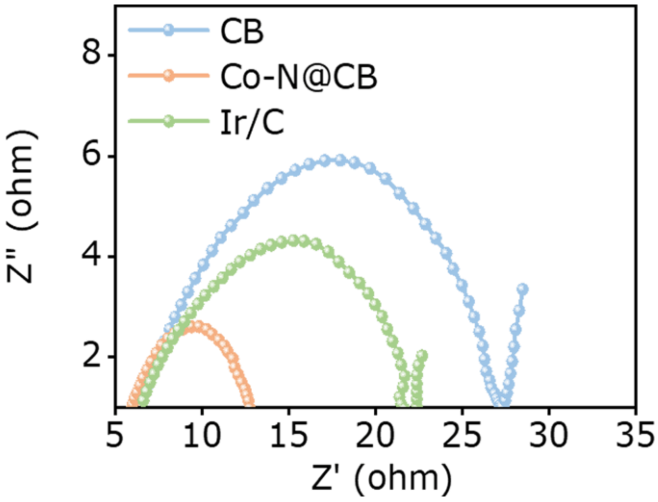

Figure S14. Nyquist curves of CB, Co-N@CB, and Ir/C.

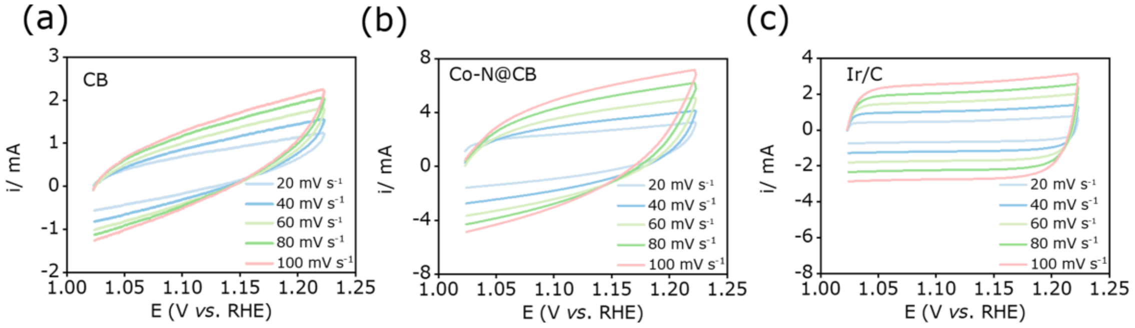

Figure S15. Cyclic voltammetry (CV) curves of (a) CB, (b) Co-N@CB, and (c) Ir/C recorded at scan rates of 20, 40, 60, 80, and 100 mV s^{-1} in the non-faradaic potential range of 1.02–1.22 V vs. RHE.

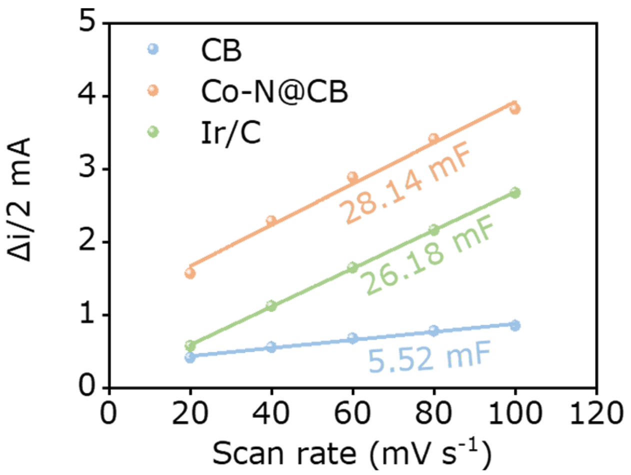

Figure S16. The summarized C_{dl} of CB, Co-N@CB, Ir/C.

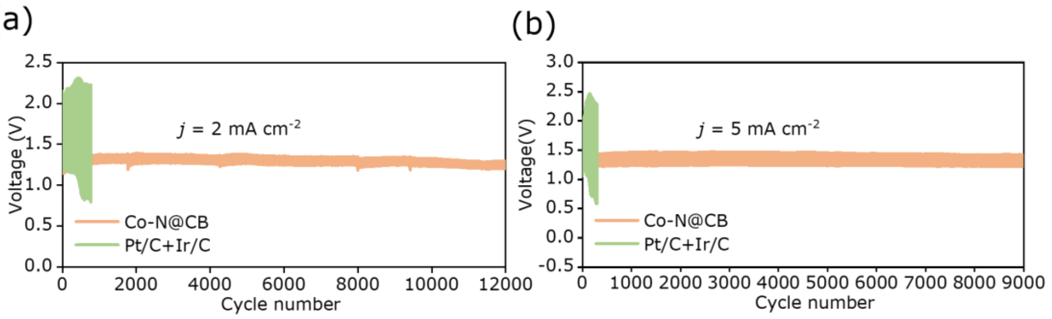

Figure S17. (a, b) long-term cycling stability of ZABs based on Co-N@CB and Pt/C + Ir/C.

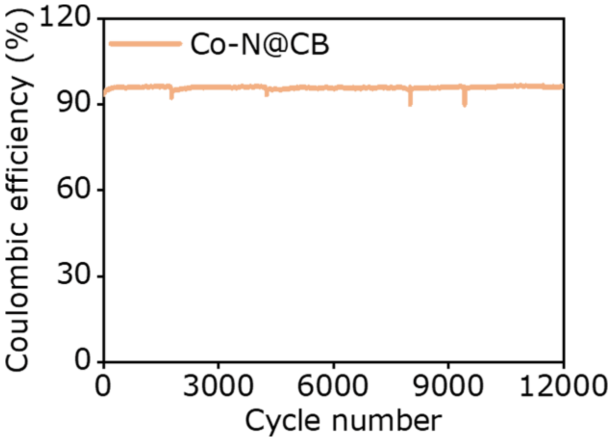

Figure S18. Coulombic efficiency of Zn–air batteries based on Co-N@CB measured at a current density of 2 mA cm^{-2} during long-term cycling.

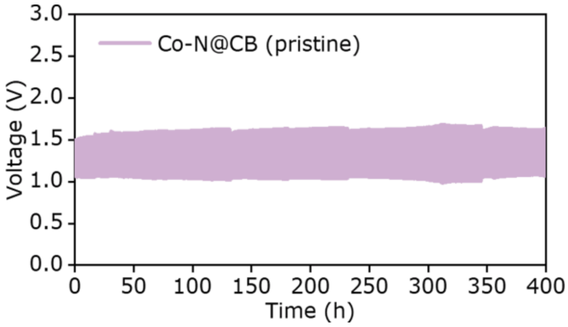

Figure S19. Long-term cycling stability of ZABs based on Co-N@CB (pristine) measured at 2 mA cm^{-2}.

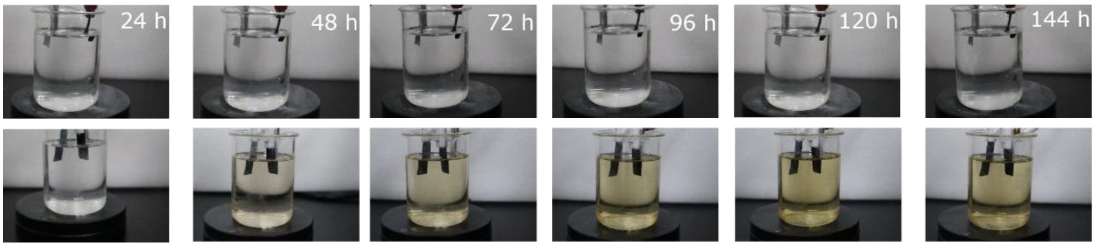

Figure S20. The sample shedding comparison diagram of ZABs based on Co-N@CB under a discharge charge current density of 5 mA cm^{-2} during the cycling test.

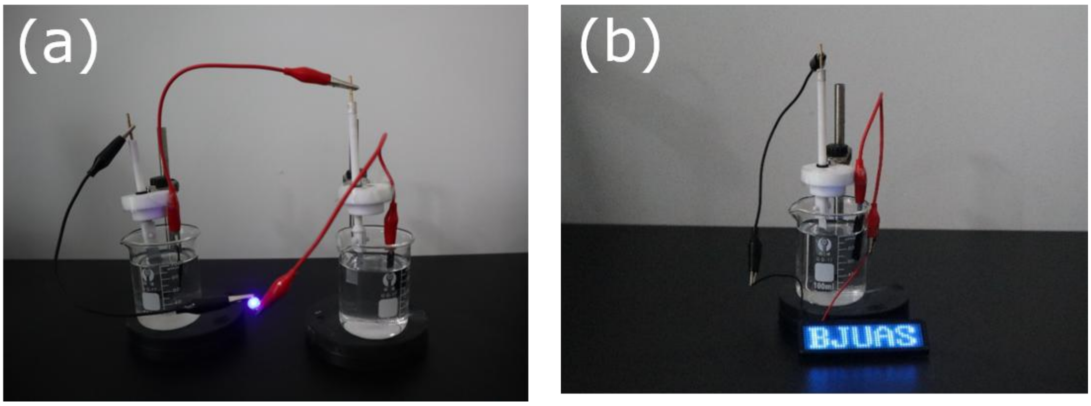

Figure S21. (a) The beads powered by ZABs assembled with Co-N@CB. (b) The light-emitting diode (LED) panel by ZABs assembled with Co-N@CB.

Section S3. Supporting tables

Table S1. Comparison of double-layer capacitance (C_{dl}) values for different electrocatalysts reported in the literature

**sample name**	**Electrolyte**	**CV Scan Rate ($mV \cdot s^{-1}$)**	**C_{dl} (mF)**	**References**
Co-N@CB	1M KOH	20-100	28.14	This work
S-NiFe LDH	1M KOH	2-200	32.4	[1]
NiFeS14@NCNM/CW	1M KOH	20-100	14.56	[2]
C-$NFF_{(5:5)}$-$LDH_{(6)}$/Fe_2O_3	1M KOH	10-100	9.55	[3]
MET-Fe/NF	1M KOH	20-100	8.19	[4]
$MoNi_4$-$Fe_{0.5}$	1M KOH	10-60	3.29	[5]
NiFe-UMNs/NF	1M KOH	20-120	3.075	[6]
Ni_3S_2/Co_9S_8@Co-NC	1M KOH	20-140	1.67	[7]
$Ba_{0.3}(SO_4)\delta W_{0.2}Ru_{0.5}O_{2-\delta}$	0.5M H_2SO_4	20-180	1.39	[8]
$CeLa_2O_3$	1M KOH	10-250	0.49	[9]
$MoSe_2$@CoAl-LDH	1M KOH	20-100	0.3	[10]

Table S2. The electrocatalytic activities of recently reported bifunctional electrocatalysts for OER and ORR.

**Catalysts**	**Half-wave potential ($E_{1/2}$, V)**	**Potential at 10 mA cm^{-2} ($E_{j=10}$, V)**	**Potential difference between $E_{1/2}$ and $E_{j=10}$ (ΔE, V)**	**References**
This Work	0.89	1.5	0.69	This work
Co_9S_8/NCNTs/CC-1	1.52	0.86	0.66	[11]
MoNP@CC	0.388	0.74	0.671	[12]
CoNi@N-CNTs/CFC	1.481	0.85	0.631	[13]
CoFeNi@N-PCF	1.55	0.85	0.7	[14]
CoFeNiFCNFs	1.64	0.92	0.72	[15]
NiFeCo-P@NC	1.52	0.793	0.727	[16]
CNT@Co-$CNFF_{50-900}$	1.61	0.87	0.74	[17]
$CoNi_2S_4$@CoNi-LDH/SSM	1.482	0.74	0.742	[18]
Co-LCFs-800	1.584	0.834	0.75	[19]
FeCo/FeCoNi@NCNTs-HF	1.608	0.85	0.758	[20]
FeCo/N-CNTs@CC	1.638	0.816	0.822	[21]
$Co_{0.25}/KMnO_4$	1.66	0.77	0.89	[22]

Reference

[1] Ma, Y.; Wang, J.; Liu, H.; Wang, L.; Sun, C.; Gong, L.; Zhang, X.; Zhu, J. Expediting corrosion engineering for sulfur-doped, self-supporting Ni–Fe layered dihydroxide in efficient aqueous oxygen evolution. *Catalysts* **2024**, *14*, 394. https://doi.org/10.3390/catal14070394

[2] Wang, Y.; Zhuang, Y.; Hu, Y.; Kong, F.; Yang, G.; Rojas, O. J.; He, M. Hollow N-doped carbon nano-mushroom encapsulated hybrid $Ni_3S_2/Fe_5Ni_4S_8$ particle anchored to the inner wall of porous wood carbon for efficient oxygen evolution electrocatalysis. *Nanoscale* **2023**, *15*, 18033–18043. https://doi.org/10.1039/d3nr03676f

[3] Zhou, Y.; Gao, J.; Ju, M.; Chen, Y.; Yuan, H.; Li, S.; Li, J.; Guo, D.; Hong, M.; Yang, S. Combustion growth of NiFe layered double hydroxide for efficient and durable oxygen evolution reaction. *ACS Appl. Mater. Interfaces* **2024**, *16*, 28526–28536. https://doi.org/10.1021/acsami.4c03766

[4] Cai, K.; Chen, W.; Wan, Y.; Chu, H.; Hai, X.; Zou, R. Self-reconstructed metal–organic framework-based hybrid electrocatalysts for efficient oxygen evolution. *Nanomaterials* **2024**, *14*, 1168. https://doi.org/10.3390/nano14141168

[5] Zhao, Y.; Zhou, P.; Li, Z.; Zhao, B.; Jiang, W.; Chen, X.; Wang, J.; Yang, R.; Zuo, C. Self-supported Fe-doped $MoNi_4$ alloy nanosheet array as a trifunctional electrocatalyst for water and urea splitting. *ACS Appl. Nano Mater.* **2024**, *7*, 14609–14620. https://doi.org/10.1021/acsanm.4c02156

[6] Zhao, X.; Yang, Y.; Liu, Y.; Shi, J.; Li, Q.; Xu, Q.; Lin, W.-F. Ultrathin Ni–Fe MOF nanosheets: efficient and durable water oxidation at high current densities. *Langmuir* **2024**, *40*, 13122–13133 . https://doi.org/10.1021/acs.langmuir.4c01065

[7] Wang, Z.; Liu, P.; Yang, C.; Zhang, Z.; Qian, Y.; Zu, Y.; Li, X.; Feng, A. Regulating coordination environment of Co single-atom through Ni/Co sulphides synergistic heterointerfaces towards efficient bifunctional oxygen electrocatalysts. *J. Mater. Chem. A* **2025**, *13*, 9974–9982. https://doi.org/10.1039/d5ta00534e

[8] Xue, Y.; Zhao, J.; Huang, L.; Lu, Y.-R.; Malek, A.; Gao, G.; Zhuang, Z.; Wang, D.; Yavuz, C. T.; Lu, X. Stabilizing ruthenium dioxide with cation-anchored sulfate for

durable oxygen evolution in proton-exchange membrane water electrolyzers. *Nat. Commun.* **2023**, *14*, 8093. https://doi.org/10.1038/s41467-023-43977-7

[9] Kaleem, M.; Shoukat, R.; Ali, A.; Naz, S.; Ahmad, A.; Khan, M. S.; Tariq, H.; Akyürekli, S.; Kaleli, M.; Alsaif, N. A. M.; *et al.* Modulation of tolerance factor and vacant oxygen holes of doped and undoped lanthanum oxides toward electrocatalytic oxygen evolution reactions: perovskites versus nonperovskites materials. *ChemCatChem* **2025**, *17*, e01562. https://doi.org/10.1002/cctc.202501562

[10] Cogal, S.; Mičušík, M.; Knotek, P.; Melanova, K.; Zima, V.; Omastová, M. Enhanced electrochemical performance of $MoSe_2$ nanosheets on CoAl-layered double hydroxide for oxygen evolution reaction. *J. Alloys Compd.* **2024**, *987*, 174173. https://doi.org/10.1016/j.jallcom.2024.174173

[11] Wu, Q.; Gao, H.; Jiang, J.; Zhao, T.; Liu, S.; Wu, C.; Xu, G.; Zhang, L. In-situ nitrogen-doped carbon nanotube-encapsulated Co_9S_8 nanoparticles as self-supporting bifunctional air electrodes for zinc–air batteries. *J. Mater. Sci. Technol* **2025**, *222*,1–10. https://doi.org/10.1016/j.jmst.2024.09.046

[12] Wang, J.; Zhang, Y.; Liao, S.; Chen, D.; Mensah, A.; Wei, Q. MoNP-doped defective carbon fibers with bark-like nanosurface as effective bifunctional electrocatalysts for Zn–air batteries. *ChemSusChem* **2024**, *17*, e202301510. https://doi.org/10.1002/cssc.202301510

[13] Shen, Y.; Yan, F.; Yang, H.; Xu, J.; Geng, B.; Liu, L.; Zhu, C.; Zhang, X.; Chen, Y. Encapsulating CoNi nanoparticles into nitrogen-doped carbon nanotube arrays as bifunctional oxygen electrocatalyst for rechargeable zinc–air batteries. *J. Colloid Interface Sci.* **2025**, *677*, 842–852. https://doi.org/10.1016/j.jcis.2024.07.227

[14] Li, M.; Zong, L.; Zhao, J.; Fan, K.; Song, F.; Zhang, Q.; Wang, Z.; Wang, L. The millisecond fabrication of medium-entropy alloy as a high-performance bifunctional electrocatalyst for ultralong-term rechargeable zinc–air batteries. *J. Alloys Compd.* **2024**, *976*, 173183. https://doi.org/10.1016/j.jallcom.2023.173183

[15] Meng, X.; Yuan, Y.; Feng, J.; Ma, C.; Sun, Y.; Zhang, J.; Pang, B.; Chen, Y.; Yu, L.; Dong, L. Design and synthesis of self-supporting FeCoNi- and N-doped carbon fibers/nanotubes as oxygen bifunctional catalysts for solid-state flexible Zn–air

batteries. *Chem. Eng. J.* **2024**, *479*, 147648. https://doi.org/10.1016/j.cej.2023.147648

[16] Kang, H.; Xiao, Y.; Feng, Q.; Su, B.; Lei, Z. Nitrogen-doped carbon self-supported NiFeCo phosphide bifunctional electrocatalysts for rechargeable zinc–air batteries. *J Energy Storage* **2025**, *111*, 115444. https://doi.org/10.1016/j.est.2025.115444

[17] Lu, T.; Xu, N.; Guo, L.; Zhou, B.; Dai, L.; Yang, W.; Liu, G.; Lee, J. K.; Qiao, J. Constructing "π–π" reinforced bridge carbon nanofibers with highly active Co-N/C@pyridine N/C@cnts sites as free-standing bifunctional oxygen electrodes for Zn–air batteries. *Adv Fiber Mater* **2024**, *6*, 1108–1121. https://doi.org/10.1007/s42765-024-00413-9

[18] Wang, Z.; Jian, J.; Wang, X.; Qiao, Y.; Wang, M.; Gao, S.; Nie, P.; Chang, L. $CoNi_2S_4$@CoNi-LDH heterojunction grown on SSM as a highly efficient trifunctional catalyst for water-splitting and Zn–air batteries. *J. Mater. Chem. C* **2023**, *11*, 16384–16389. https://doi.org/10.1039/d3tc03336h

[19] Wang, Y.; Gan, R.; Shao, X.; Dai, B.; Ma, L.; Yang, J.; Shi, J.; Zhang, X.; Ma, C.; Jin, Z. Co/CoO hetero-nanoparticles incorporated into lignin-derived carbon nanofibers as a self-supported bifunctional oxygen electrocatalyst for rechargeable Zn-air batteries. *J. Colloid Interface Sci.* **2025**, *682*, 934–945. https://doi.org/10.1016/j.jcis.2024.12.035

[20] Wang, Z.; Ang, J.; Zhang, B.; Zhang, Y.; Ma, X. Y. D.; Yan, T.; Liu, J.; Che, B.; Huang, Y.; Lu, X. FeCo/FeCoNi/N-doped carbon nanotubes grafted polyhedron-derived hybrid fibers as bifunctional oxygen electrocatalysts for durable rechargeable zinc–air battery. *Appl. Catal. B: Environ.* **2019**, *254*, 26–36. https://doi.org/10.1016/j.apcatb.2019.04.027

[21] Li, Z.; Yang, H.; Sun, H.; Liang, S.; Kou, S. Highly nitrogen-doped carbon nanotube nanoarrays as self-supported bifunctional electrocatalysts for rechargeable and flexible zinc–air batteries. *ACS Sustainable Chem. Eng.* **2021**, *9*, 4498–4508. https://doi.org/10.1021/acssuschemeng.0c08727

[22] Zheng, X.; Moreno Zuria, A.; Mohamedi, M. Free-Standing Tunnel-Structured MnO2 Nanorods-Doped with Nickel and Cobalt Cations as Bifunctional Electrocatalysts for Zn–Air Batteries. *Adv. Mater. Technol.* **2023**, *8*, 2301142. https://doi.org/10.1002/admt.202301142
